# Human Mesenchymal Stem Cells Self-Renew and Differentiate According to a Deterministic Hierarchy

**DOI:** 10.1371/journal.pone.0006498

**Published:** 2009-08-04

**Authors:** Rahul Sarugaser, Lorraine Hanoun, Armand Keating, William L. Stanford, John E. Davies

**Affiliations:** 1 Institute of Biomaterials and Biomedical Engineering, University of Toronto, Toronto, Ontario, Canada; 2 Princess Margaret Hospital, Toronto, Ontario, Canada; Tufts University, United States of America

## Abstract

**Background:**

Mesenchymal progenitor cells (MPCs) have been isolated from a variety of connective tissues, and are commonly called “mesenchymal stem cells” (MSCs). A stem cell is defined as having robust clonal self-renewal and multilineage differentiation potential. Accordingly, the term “MSC” has been criticised, as there is little data demonstrating self-renewal of definitive single-cell-derived (SCD) clonal populations from a mesenchymal cell source.

**Methodology/Principal Findings:**

Here we show that a tractable MPC population, human umbilical cord perivascular cells (HUCPVCs), was capable of multilineage differentiation *in vitro* and, more importantly, contributed to rapid connective tissue healing *in vivo* by producing bone, cartilage and fibrous stroma. Furthermore, HUCPVCs exhibit a high clonogenic frequency, allowing us to isolate definitive SCD parent and daughter clones from mixed gender suspensions as determined by Y-chromosome fluorescent in situ hybridization.

**Conclusions/Significance:**

Analysis of the multilineage differentiation capacity of SCD parent clones and daughter clones enabled us to formulate a new hierarchical schema for MSC self-renewal and differentiation in which a self-renewing multipotent MSC gives rise to more restricted self-renewing progenitors that gradually lose differentiation potential until a state of complete restriction to the fibroblast is reached.

## Introduction

Tissue resident progenitors that give rise to connective tissue cells *in vitro* have been isolated from a variety of tissues including bone marrow (BM) [1], [2], [3], [4], fat [5], [6], muscle [7], [8], placenta [9], umbilical cord [10], [11], [12], [13], [14] and fetal liver [15]. These cells are generally thought to be resident in the perivascular compartment of these tissues [16], [17], [18], [19], [20]; and are commonly called “mesenchymal stem cells” (MSCs) [21]. While it is generally accepted that these cells include rare stem cells [22], [23], [24], the term “MSC” has been criticised [25], since the definition of a stem cell assumes that these progenitors have clonal self-renewal and multilineage differentiation potential. To date, assaying these two properties in MSCs has been extremely challenging due to the low frequencies of clonogenic progenitors found in most tissues (e.g., mesenchymal clonogenic frequency in human BM is 1∶10^4^ to 1∶10^6^ depending on age).

To date only two studies have succeeded in isolating definitive single-cell-derived (SCD) clonal populations by deposition of single human mesenchymal cells into 96-well plates by fluorescence activated cell sorting (FACS) [26]. However, neither study concurrently assayed each clone for more than one phenotype, or determined whether each clone had self-renewal capacity by producing equipotent progeny. While other investigators [27] did analyze ‘clones’ for bi- and trilineage (bone, cartilage and adipose) differentiation capacity, they utilized limiting dilution [27], [28], [29] by seeding 10^3^–10^5^ cells/culture well to generate fibroblast colonies (CFU-F). Thus, these studies did not provide definitive evidence of SCD clonality. Other studies attempted to overcome this issue by seeding cells at very low densities to isolate their colonies [30], [31], but did not verify the single cell origin of their populations. Furthermore, none of these studies determined whether the mesenchymal cells isolated maintained multipotential capacity *in vivo*. Thus, to date, demonstration of a definitive MSC has remained refractory to robust experimental evidence [32].

To overcome these limitations, we utilized populations of non-alloreactive [33] human umbilical cord derived mesenchymal progenitors (HUCPVCs) [10], [34], harvested from a total of 57 cords, that maintain a high clonogenic frequency *in vitro* to isolate definitive SCD clones. We found that populations of HUCPVCs undergo multilineage differentiation and contribute to repair of osteochondral defects *in vivo*. We next isolated definitive parent and daughter clones from mixed gender suspensions and then assayed these clones for their ability to differentiate into one or more of five mesenchymal lineages: bone, cartilage, fat, muscle and fibrous tissue. Our results demonstrated that these SCD clones maintained extensive self-renewal capacity *in vitro* and clonally produced daughter populations of cells with quinti-, quadri-, tri-, bi- and unilineage potential. Analysis of the differentiation trajectories of the parent and daughter clones uncovered a new hierarchical schema for MSC self-renewal and differentiation.

## Results

### Multipotential HUCPVC mesenchymal progenitors are maintained in long-term culture

**Figure 1:**
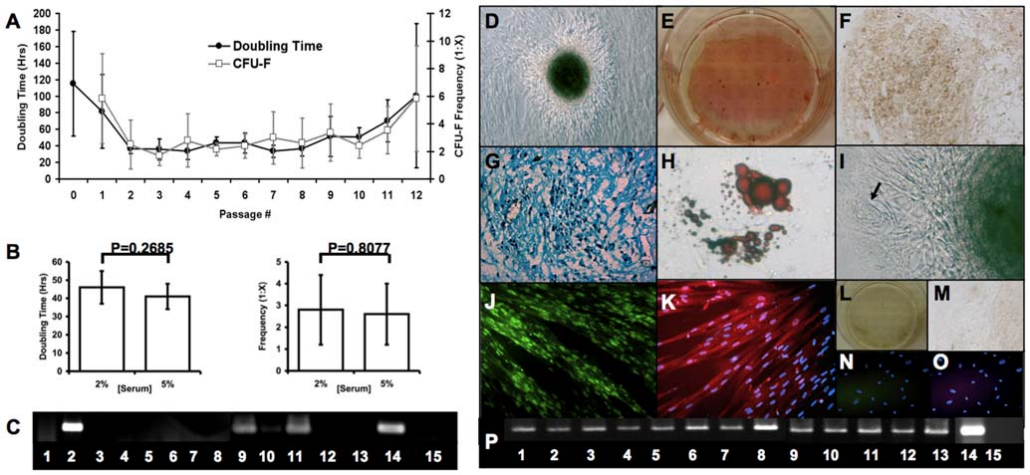
HUCPVC populations exhibit rapid proliferation, high clonogenicity and multipotential capacity *in vitro*. HUCPVC populations maintained stable proliferation and clonogenic frequency from passage 2 through 9 (A, n = 11 different cords), with no significant difference in either parameter at passage 5 (B, n = 7 different cords). Data are represented as mean +/− s.d. In standard culture conditions, HUCPVCs expressed Collagen IA1, Desmin and MyoD [lanes 2, 9 and 11 respectively, all other lanes as represented in (P)] as determined by RT PCR (C). HUCPVCs could further differentiate into bone, cartilage, adipose and muscle *in vitro*. With induction, bone nodules were observed in culture (D) that stained with Von Kossa (black), and were surrounded by alkaline phosphatase-high expressing cells (E). Cartilage pellet cultures of HUCPVCs expressed collagen II (F) and glycosaminoglycans that stained with Alcian blue (G). HUCPVC-derived adipocytes stained with Oil Red O (H) and occasionally formed spontaneously (arrow) in association with bone nodules (I). Myogenically-induced HUCVPCs expressed high levels of MyoD (J) and fast skeletal myosin light chain (FSMLC) (K) in multinucleated HUCPVC myotubes. Negative controls are non-induced cells stained with Von Kossa and alkaline phosphatase (L), and secondary-only antibody staining for collagen II (M), MyoD (N) and FSMLC (O) stained cultures. (Field widths: D,F = 628 µm; E,L = 3.5 mm; G,J,K,N,O = 315 µm; H,I = 86 µm). RT PCR analysis (P) demonstrated upregulation of the following lineage-specific genes: Runx2 (1), collagen IA1 (2), osteopontin (3), osteocalcin (4), Sox9 (5), collagen II (6), lipoprotein lipase (LPL) (7), aggregan (8), MyoD (9), Myf5 (10), desmin (11), myosin heavy chain (MHC) (12), FSMLC (13), GAPDH (14) and RT- control (15). (Cells from 9 cords, some of which are common to other assays, were employed in these functional phenotypic and gene expression data).

We, and others, have previously shown that HUCPVC populations express CD44, CD73, CD90, CD105, and CD106 but do not express CD45 or CD34 [10], [13], [34]. Furthermore, we had previously observed that HUCPVCs exhibit a doubling time of 20 hours when cultured in 15% fetal bovine serum (FBS) [10]. Here, we utilized serum concentrations of 5% or 2% which increased the doubling time (41±7 and 46±9 hours respectively), but also maintained a consistent doubling time and clonogenic frequency of approximately 1∶3 (1: [2.6±1.4] and 1: [2.8±1.6] respectively) for more than 10 passages (Figure 1A,B) unlike adult bone marrow-derived mesenchymal cells [35], [36].

We next tested whether bulk HUCPVC populations were capable of differentiating into five mesenchymal lineages *in vitro*: myogenic (M), adipogenic (A), chondrogenic (C), osteogenic (O) and fibroblastic (F). Utilizing serum concentrations ranging from 2–3%, HUCPVCs were assayed at passage 3 (P3) (∼10 population doublings) and P6 (∼15 doublings) for differentiation capacity into these lineages. Results were the same for both P3 and P6 cells. In non-induced conditions (Figure 1C), HUCPVCs expressed collagen IA1, desmin and low levels of MyoD, characteristic of their ‘myofibroblastic’ [37], [38] phenotype. In osteogenic culture conditions (Figure 1D,E,P), expression of Runx2, collagen IA1, osteopontin and osteocalcin were upregulated. The cells changed from a fibroblastic to a more tessellated osteoblast-like morphology, and formed bone nodules by elaborating mineralized bone matrix as evidenced by the presence of positive tetracycline staining (data not shown), alkaline phosphatase and positive Von Kossa staining. In chondrogenic culture conditions (Figure 1F,G,P), upregulation of Sox9, collagen II and aggrecan were observed. The cells elaborated collagen II matrix and sulphated glycosaminoglycans, verified by immuno-histochemistry and Alcian blue staining respectively. HUCPVCs could also be induced to differentiate into lipoprotein lipase-expressing adipocytes (Figure 1H,I,P) in culture that stained with oil red O, occasionally forming spontaneously in association with bone nodules. Although we did not quantify the relative expression of each of these phenotypes, Baksh et al [34] have determined the relative quantification under osteogenic, chondrogenic and adipogenic induction. As non-induced HUCPVCs express myogenic markers including desmin, vimentin and alpha smooth muscle actin [37], [38], [39], myogenically-induced cells (Figure 1J,K,P) were analyzed for differential expression of MyoD and fast skeletal myosin light chain (FSMLC). Non-induced cells expressed low levels of MyoD, but no FSMLC, while induced cells formed long multinucleated myotube-like structures that expressed high levels of MyoD and FSMLC. This was confirmed by RT-PCR analysis, in which upregulation of MyoD, Myf5, desmin, myosin heavy chain (MHC), and FSMLC were observed.

### HUCPVC populations contribute to multilineage repair *in vivo*

**Figure 2:**
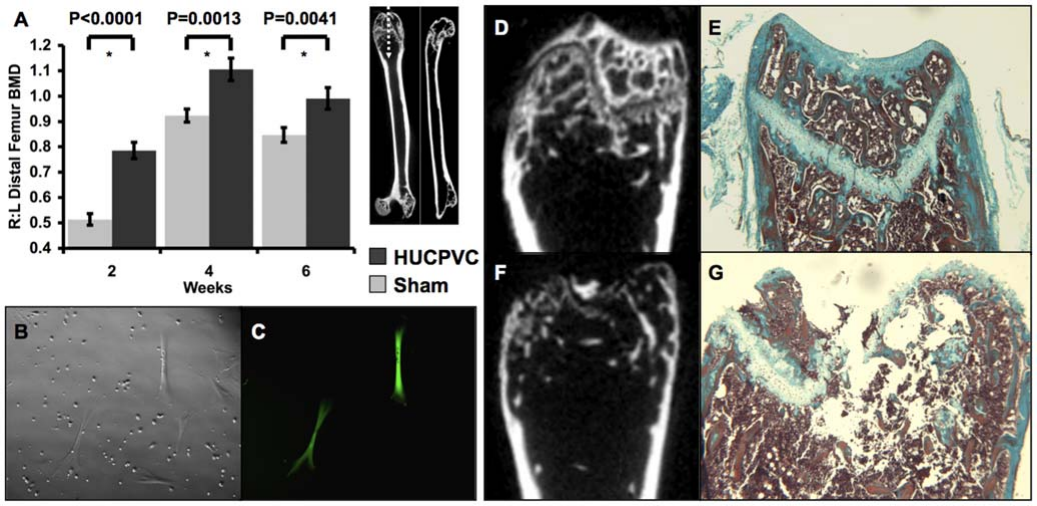
HUCPVCs survive and regenerate damaged mesenchymal tissues *in vivo*. HUCPVCs were synthetically active and survived at least six weeks *in vivo* when implanted into the intrafemoral space of NOD-scid mice as observed by the presence of GFP-labeled cells among mouse cells flushed from the marrow into culture (B,C). When the distal ends of the femurs were analyzed by micro-computed tomography (µCT), it was determined that these cells produced significantly more bone mineral density (BMD) than sham controls at 2,4 and 6 weeks (A, values are means±s.d.). µCT analysis and Masson's Trichrome stained longitudinal sections of injected femurs showed that HUCPVCs induced significantly more repair of bone and cartilage at 2 weeks (D,E) compared to sham controls (F,G). HUCPVC populations from a total 6 different cords were employed to generate this data and that shown in Figure 3.

**Figure 3:**
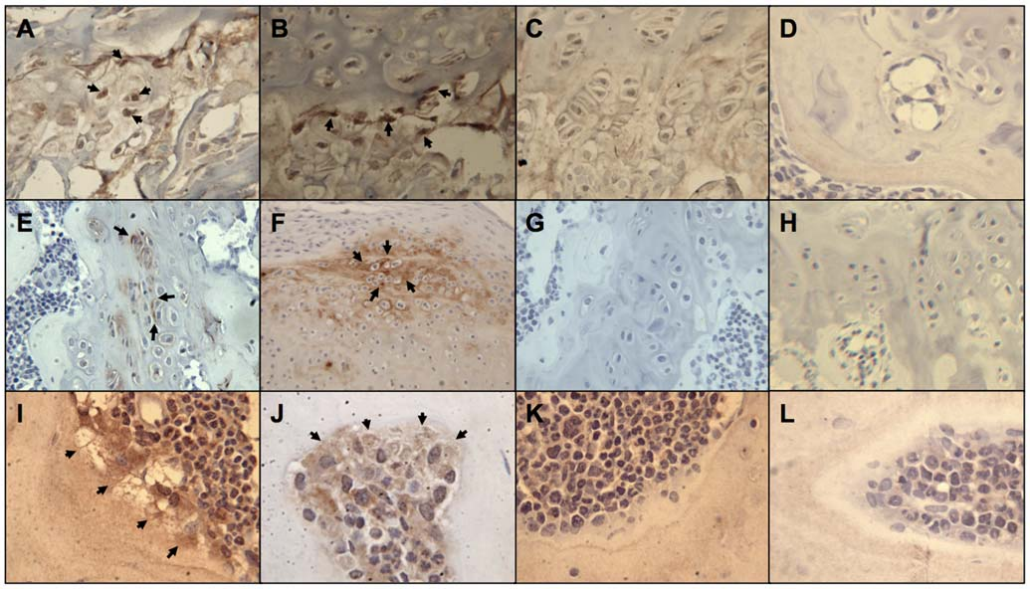
HUCPVCs display multipotential capacity *in vivo*. When labeled with HuNu (human nuclear antigen), human cells (arrows) were observed in the growth plate of the distal femurs (A,B field widths = 140 µm). These cells were associated with the presence of human-specific collagen II (arrows) in the extracellular matrix surrounding individual chondrocytes (E,F field widths = 140 µm and 212 µm respectively). Human-specific osteocalcin was also observed on the osteoid of newly forming bone. Arrows indicate human-specific osteocalcin was present surrounding individual cells that produced a collagen-like matrix on the surface of the bone (I,J field widths = 110 µm). No staining was observed in sham-injected femurs or negative controls for HuNu (C,D), collagen II (G,H) or osteocalcin (K,L) respectively (same field widths as experimentals).

To determine the capacity of HUCPVC populations to contribute to repair *in vivo* we utilized a transplantation model originally developed to assay human hematopoietic stem cell repopulation [40]. HUCPVCs that had been infected with lentivirus encoding eGFP driven by a constitutive promoter were injected into the femoral marrow cavity of NOD-scid mice. The tissue was analyzed at 2, 4 and 6 weeks post-transplantation. After 2 weeks, there was significantly more healing of bone and cartilage in the HUCPVC-injected femora than in contralateral sham-injected controls (Figure 2A,D-G). By flushing the marrow and recovering culture-adherent eGFP-labeled cells, we showed that HUCPVCs survived at least 6 weeks *in vivo* (Figure 2B,C). By 4 and 6 weeks, complete autologous repair was achieved as there did not appear to be any gross difference between the experimental and control femurs. When the distal region of each femur was analyzed by micro CT (Figure 2A), the BMD difference was larger at all three time points, illustrating greater mineralized bone formation in the HUCPVC-transplanted femurs. To determine whether the HUCPVCs contributed to healing by becoming synthetically active, we labeled them with monoclonal antibodies for human-specific osteocalcin, collagen II and PPARγ [41]. As the eGFP label had been eliminated by decalcification of the tissues, we also labeled sections with HuNu, an antibody that specifically labels human nuclei and not those of mice. Figure 3A,B illustrates HuNu-labeled cells in the femoral growth plate that were associated with human-specific collagen II staining (Figure 3E,F). Anti-human osteocalcin labeled in the cytoplasm of some cells as well as within the newly formed bone matrix (Figure 3I,J). PPARγ staining was non-specific. As expected, no positive staining was observed in sham injected femurs (Figure 3C,G,H) or negative controls (Figure 3D,H,L). Interestingly, the majority of cells in the HUPVC-injected femurs did not label with the anti-human antibodies, suggesting that although the HUCPVCs did differentiate and produce some tissue, they may have had a larger role in recruiting and directing the mouse mesenchymal progenitors to repair the damaged tissue. This is strikingly evident in the cartilage repair, as the mouse cells rapidly produced new collagen II distal to the more slowly produced human collagen II (Figure 3E). Together, these data demonstrate that HUCPVCs have multilineage differentiation potential *in vitro* and, after transplantation, differentiate into at least two distinct lineages that contribute to tissue repair *in vivo*.

### Traditional mesenchymal cell seeding methods do not isolate definitive SCD clones

**Figure 4:**
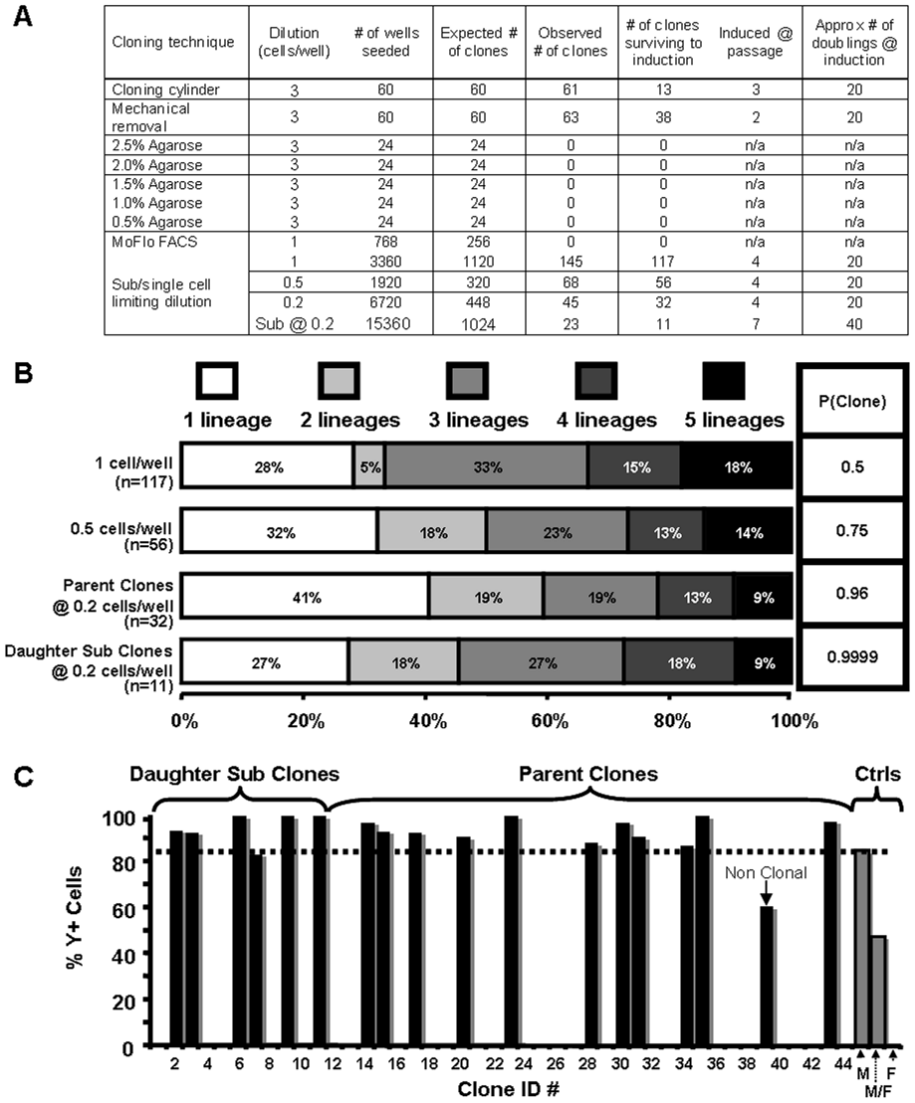
Clonally pure HUCPVC populations can be isolated by rigorous cell seeding. Five different methods were used in an attempt to generate definitive single-cell-derived (SCD) clones from a total of 12 cords, some of which were employed in other assays, (A). Clonal colonies generated by seeding mixed male and female suspensions of HUCPVCs at 1, 0.5, and 0.2 cells/well, the latter of which were sub-cloned (daughters) at 0.2 cells/well, displayed increasing probabilities of SCD isolation along with differential multipotential capacities (B). When analyzed by fluorescent in situ hybridization (FISH) with a Y-chromosome-specific probe, all the daughters (clones 1–11) as well as all but one (clone 39) of the parent clones (clones 12–44) seeded at 0.2 cells/well were found to be single gender derived, and accordingly determined to be SCD clones (C).

Having established low serum-dependent culture conditions that provided a clonogenic frequency of>1∶3, and determined that bulk HUCPVC populations could differentiate into multiple mesenchymal lineages both *in vitro* and *in vivo*, we used five different methods to isolate SCD HUCPVC populations (Figure 4A, and see Supplementary Methods S1) and assayed their differentiation capacity into the five mesenchymal lineages (Figure 4B). For clonal expansion, approximately 20 cell doublings from a single originating cell were required to provide ∼10^6^ cells needed for lineage analysis.

The cloning cylinder has been the traditional method utilized in mesenchymal literature to isolate limiting dilution-derived mesenchymal colonies. Based on a 1∶3 CFU-F frequency of HUCPVCs, limiting dilution seeding of 3 cells per well, by definition, yielded 100% colony formation. This method provided a relatively poor yield of HUCPVC colonies that survived the first sub-culture (∼20%). Use of a novel ‘inverse’ method of isolating these colonies by removing any and all cells around the colony, improved the yield to ∼60% because the cells could be grown to sub-confluence before sub-culture. We hypothesized that by culturing these cells on agarose, it would be easier to pick the growing colonies for subculture, but after several attempts with different concentrations of agarose, the cells invariably migrated to the bottom of the well and colonies could not be ‘picked’ for sub-culture. Nevertheless, as 3 cells were required to generate colonies for isolation in each of these techniques, it could not be definitively determined that these colonies were truly SCD [42]. As a result, a MoFlo FACS was used to seed single cells into individual wells of 96-well plates. After four attempts at seeding a total of 768 wells, no colonies developed. Lack of cell proliferation may be attributed to the cumulative time required to remove the cells from the culture surface to put them into suspension, the shear forces exerted on the cells by the FACS machine, and a lack of paracrine signalling required to stimulate cell survival and proliferation.

Finally, a single-cell-dilution of HUCPVCs was used to seed individual HUCPVCs. A total of 3,360 wells were seeded at a dilution of 1 cell/well that yielded 117 clones (3.48%) surviving to induction at P4 (∼20 population doublings). As the probability that each of these clones was single-cell-derived was only 0.5, a further dilution of 0.5 cells/well was used with an associated probability of 0.75 and yield of 2.92%. The mean CFU-F frequency of the clones was 1: [3.8±1.9] (Figure 4A), with no significant difference (P = 0.391) between clones derived from each seeding density. This value was not significantly different (P = 0.125) from the 1: [2.6±1.4] frequency seen in bulk HUCPVC cultures (Figure 1A), suggesting that the progenitor pool was maintained upon cloning. Lineage analysis of these ‘clones’ (Figure 4B) illustrated that as the seeding density decreased, so did the number of multipotential ‘clones.’ There appeared to be an increase in unipotential ‘clones’ and a decline in tri-, quadri- and multipotential ‘clones’ as the seeding density became more rigorous. This observation suggested that ‘clones’ isolated by seeding at 1 or 0.5 cells/well were not SCD colonies.

### Rigorous sub-single cell seeding of mixed gender suspensions of HUCPVCs produce definitive SCD clones

We realized that by seeding cells at a dilution of 0.2 cells/well (1 cell per 5 wells) the probability of isolating SCD clones increased to a statistically significant 0.96 (Figure 4B). To make this strategy even more rigorous, we mixed equal numbers of male and female cells prior to seeding 0.2 cells/well (1 cell per 5 wells) into 96-well plates, which would enable genotyping of clones. From a total of 6,720 wells seeded, 45 clonal populations were produced, 32 of which survived expansion to ∼10^6^ cells. These 32 clones were re-seeded at the same dilution of 0.2 cells/well into 480 wells for each clone, which produced 23 clonal daughter populations, 11 of which survived expansion to ∼10^6^ cells. The probability that these 11 daughter sub-clones were single-cell derived was 0.9999 (Figure 4B). Double blind Y-chromosome FISH analysis was then used to definitively confirm the single cell origin of the 32 parental and 11 daughter clones (Figure 4C). Positive, negative and mixed FISH controls determined≥85% or≤0% thresholds for determination of single-cell-derivation. All clones and sub-clones, except one, had a signal above the threshold for single-cell determination. We analyzed 22–54 cells within each of the 32 clones and determined that the probability that each was formed from more than one cell between 10^−7^ and 10^−17^. Thus, 42 of the 43 isolated clones were determined to be definitively SCD populations (parent and daughter clone ID's are shown in Supplementary Figure S1). Importantly, our finding that single-gender clones arose from a mixed gender cell suspension suggests that only an exceptionally rigorous strategy to isolate single cells, such as the one we used, could generate reliable clonal data. This is further emphasized in the generation of a non-clonal population by this method (Figure 4C: clone 39), that calls into question the reliability of clonal data produced by any less rigorous methodology.

### Definitive multipotential SCD mesenchymal clones self-renew in culture

**Figure 5:**
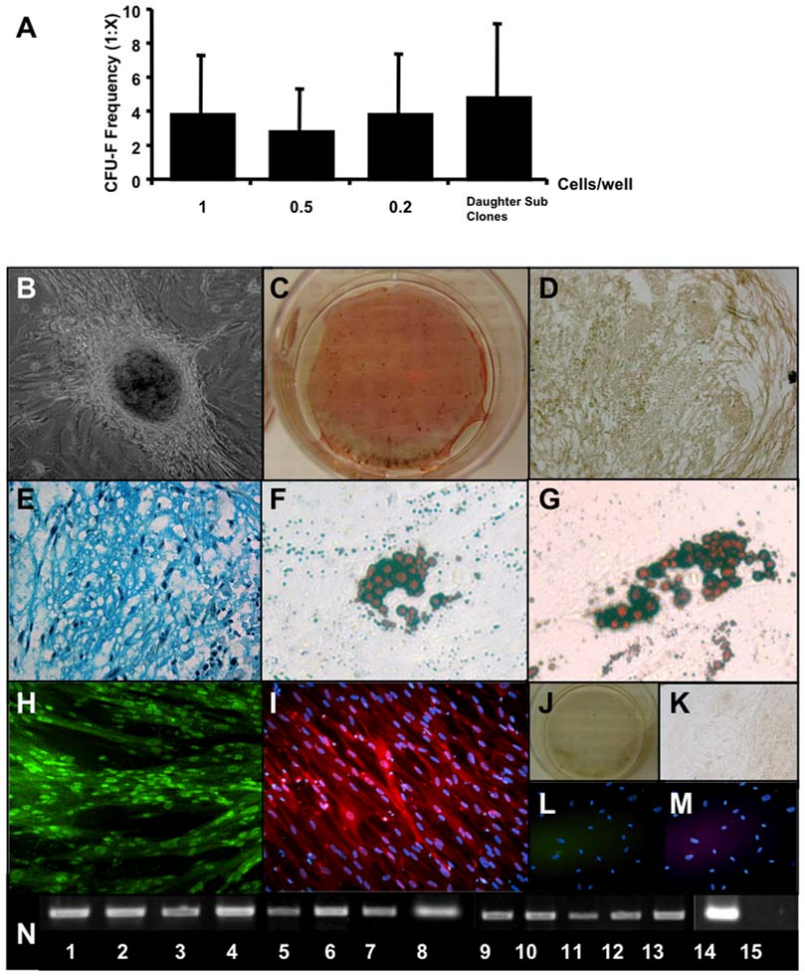
Clonally pure HUCPVC populations display multipotent capacity *in vitro*. CFU-F frequencies of HUCPVCs derived by seeding at 1, 0.5, and 0.2 cells/well, the latter of which were sub-cloned (daughters) at 0.2 cells/well, were not found to be significantly different from each other (A). Clone 11 (B-N) demonstrated identical capacity to its parent (clone 35), maintaining the ability to differentiate into all five lineages assayed. Under induction, bone nodules were observed in culture (B) that stained with Von Kossa (black), and were surrounded by alkaline phosphatase-high expressing cells (C). Cartilage pellet cultures of HUCPVCs expressed collagen II (D) and glycosaminoglycans that stained with Alcian blue (E). HUCPVC-derived adipocytes stained with Oil Red O (F,G). Myogenically-induced HUCVPCs expressed high levels of MyoD (H) and FSMLC (I) in multinucleated HUCPVC myotubes. Negative controls were uninduced cells stained with Von Kossa and alkaline phosphatase (J), and secondary-only antibody staining for collagen II (K), MyoD (L) and FSMLC (M) stained cultures. (Field widths: B,D,K = 628 µm; C,J = 3.5 mm; E,H,I,L,M = 315 µm; F,G = 86 µm). RT-PCR analysis (O) demonstrated upregulation of the following lineage-specific genes: Runx2 (1), collagen IA1 (2), osteopontin (3), osteocalcin (4), Sox9 (5), collagen II (6), LPL (7), aggregan (8), MyoD (9), Myf5 (10), desmin (11), MHC (12), FSMLC (13), GAPDH (14) and RT negative control (15). (MACOF represents a clone that was able to differentiate into all 5 lineages assayed). HUCPVC harvests from 27 different cords were employed to generate this data.

As the CFU-F frequencies of parent and daughter SCD clones were not significantly different from each other (1: [4±3.36] and 1: [5±4.15], respectively, Figure 5A), this was the first line of evidence suggesting that the parent clones may have been self-renewing and maintaining their progenitor pool. This self-renewal ability was further supported by the observation that the daughter clones maintained the potential to differentiate along more than a single lineage after>40 cell doublings.

**Figure 6:**
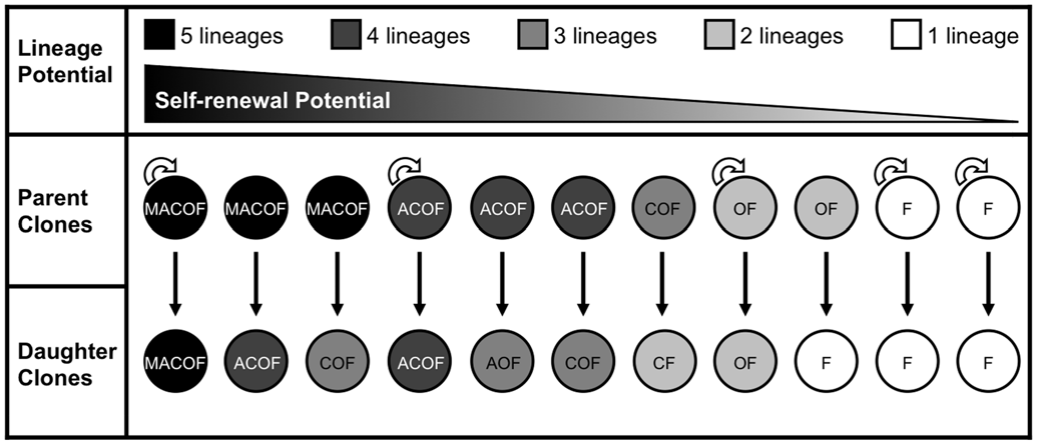
Clonally pure HUCPVC populations display differential self-renewal capacity *in vitro*. When the differentiation capacities of the 11 daughter clones were compared to those of their parents, it was found that they either maintained equipotency (self-renewing clones) or forfeited the ability to differentiate into one or more lineages (non-self-renewing clones). (Self-renewing clones are represented with a semi-circular arrow and non-self-renewing clones without a semi-circular arrow).

The 11 surviving daughter clones, when assessed for multilineage capacity (Figures 4B, 6, Supplementary Figure S1), demonstrated that they were the progeny of either a self-renewing parent of the same differentiation potential, or a parent which had forfeited one or more of its lineages to give rise to a more restricted daughter. These phenomena were most evident when observing the differentiation patterns of the first three quintipotential MACOF parental clones. The first MACOF parental clone self-renewed in culture by producing a MACOF daughter, while the remaining two differentiated by giving rise to more restricted ACOF and COF daughters, forfeiting their myogenic and myogenic/adipogenic potentials respectively. This pattern was repeated in the remaining more-restricted clones. The three quadripotent ACOF parental clones gave rise to ACOF, AOF and COF daughters, illustrating self-renewal by generation of the former and differentiation by restriction into the latter two; while the single tripotential COF parent also did not self-renew, as it forfeited its osteogenic potential by producing a bipotential CF daughter. Of the two bipotential OF parental clones, only one self-renewed into an OF daughter, while the other forfeited its osteogenic capacity by giving rise to a unipotential CFU-F. Finally, the two remaining unipotential CFU-F parental clones self-renewed in culture by producing CFU-F daughters. We further observed that, of the 21 parental clones that did not survive expansion to lineage analysis of daughter clones, 12 did survive the sub-cloning process, while 9 did not (Supplementary Figure S1). Interestingly, 8 out of 12 of clones that did survive sub-cloning (but not expansion) had potential for more than one lineage. This illustrates that, although they too did eventually clonally exhaust in culture, they were relatively more robust than the unipotent CFU-Fs.

These data, which illustrate daughter SCD clones, having undergone ∼40 population doublings, derived from equal or greater potential SCD parents, is the first reported evidence of self-renewing multipotent mesenchymal cells *in vitro*. One striking observation when the lineages are observed individually is the absence of the adipogenic lineage in the bipotential clones that possessed either an osteogenic or chondrogenic capacity. The former theoretically arises at twice the frequency of the latter, and is supported by our previously published data in which we observed a 1∶1 ratio of uncommitted to committed CFU-O progenitors in HUCPVC culture [10]. The tripotential clones illustrated that, when differentially expressed with osteogenic potential, the chondrogenic and adipogenic capacities were exclusive of each other. When these two lineages were expressed together, it was only in the quadri- and quintipotential clones. Interestingly, the myogenic lineage was only expressed in the quintipotential clone, suggesting that it is the first lineage forfeited when a multipotential MSC differentiates.

## Discussion

The MSC concept is predicated upon the elegant studies done in the hematopoietic system in which several investigators showed that a single prospectively isolated hematopoietic stem cell (HSC) could repopulate the hematopoietic system of a living recipient, and that when re-isolated from this primary recipient, this cell's progeny could reconstitute the hematopoietic system of a secondary living recipient [43], [44], [45]. Rather than the irradiation-induced injury in these hematopoietic studies, we have created a physical injury by needle marrow ablation. In fact, we have previously demonstrated that mice with defective self-renewal of multipotent mesenchymal cells developed type II osteoporosis [46], [47], suggesting that a bonafide MSC does exist within the mesenchymal compartment. However, the dearth of markers for prospective isolation of these cells has made interrogation of the MSC concept refractory to experimental validation. In an attempt to overcome this, numerous studies [4], [26], [27], [28], [30], [31], [48] have tried to retrospectively analyze the multipotential capacity of clonally-derived mesenchymal cells. There are four major caveats of these studies. First, as prospective isolation of multipotential MSCs still eludes us, and retrospective analysis is a valid alternative strategy, most investigators have limited their analysis up to three mesenchymal phenotypes: bone, fat and cartilage. While these three lineages are the most established, it is commonly accepted that several other mesenchymal lineages exist including muscle, tendon, ligament and stromal tissues [21], [49]. As the latter three could be grouped into the “fibroblastic lineages,” it then becomes clear that for full retrospective analysis of mesenchymal differentiation, five lineages must be assayed: bone, cartilage, fat, muscle and fibrogenic. The second, and most elusive issue is that of clonality. Based on Friedenstein's early work [50], [51], it was originally assumed that limiting dilution (i.e. the minimum number of cells required to produce a single colony) of mesenchymal cells could provide clonal colonies. More recently, it has been shown that ‘clonal’ cultures of neural stem cells are highly motile and prone to aggregation [42]. As mesenchymal cells are highly motile in culture, this calls into question the literature in which clonal isolation was performed by seeding more than one cell in a culture well [4], [29]. Thus, only FACS single cell deposition or sub-single cell seeding with retrospective analysis of clonal purity could be used to isolate definitive SCD clones. Third, it has yet to be definitively demonstrated either *in vitro* or *in vivo* that one of these clonally derived mesenchymal populations is able to self-renew by producing equipotent progeny either *in vitro* or *in vivo*. The final and most difficult problem is that, unlike the rigor within HSC literature, rigorous clonal mesenchymal transplantation studies have been elusive for the field.

**Figure 7:**
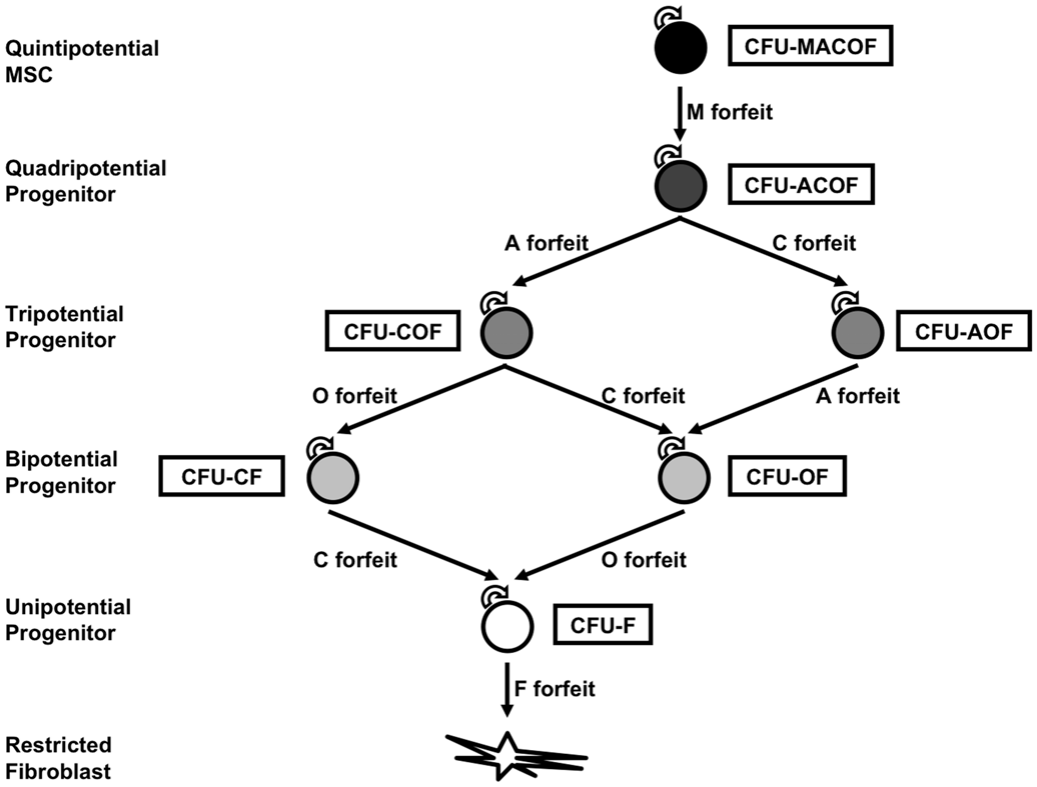
Hierarchy of MSC differentiation.

In this study, we tackled the first three problems and made inroads to address the clonal *in vivo* studies. We have described that a multipotent population of mesenchymal cells that maintain differentiation capacity *in vivo* and, due to their high clonogenic frequency, can be rigorously isolated as SCD populations in culture that in turn can generate daughter SCD populations, both of which can be analyzed for five distinct mesenchymal lineages. These *in vitro* functional phenotypic data determine a hierarchical structure for the mechanism underlying MSC differentiation (Figure 7) in which a self-renewing multipotent MSC (CFU-MACOF) gives rise to more restricted self-renewing progenitors that gradually lose differentiation potential until a state of complete restriction to the fibroblast is reached. Importantly, the sum total of all these progenitors provides the 1: [2.6±1.4] clonogenic frequency observed in our HUCPVC cultures. Other hierarchical structures of MSC differentiation have been proposed. Aubin [52] postulated that MSCs give rise to either a CFU-OC or CFU-OA bipotential progenitor and that CFU-A and CFU-C are differentially forfeited to result in the osteogenic capacity required for bone formation. Similarly, Muraglia and colleagues [27] concluded that bone represents the default in the mesenchymal pathway. However, neither group included either the myogenic or fibroblast lineages in their analyses and, as our results demonstrate, it is the latter that is the default lineage in the mesenchymal hierarchy.

The perivascular niche [20] has been shown to be the source of mesenchymal progenitors in many organs [16], including bone marrow [53]. It was recently shown [29] that the CD146^+^ osteo-progenitor cells in human marrow predominantly associate with vessels as ‘adventitial reticular cells’ and promote hematopoietic supportive stroma. While occupying their niche, perivascular cells remain quiescent but in most organs give rise to daughter cells that preferentially differentiate into the stromal fibroblasts of the organ, and by-pass other differentiation pathways presumably through signaling events that prevent the differentiation of multipotent MSC into the other restricted progenitors. Thus, we hypothesize that the primary responsibility of HUCPVCs in situ is to maintain their niche's stromal tissue by differentiating into the myofibroblasts that elaborate the extracellular matrix of the umbilical cord. However, when the homeostasis of this, or any other, perivascular niche is disturbed during growth or by injury, or these resident MSCs are removed from their natural environment, they will be provided with the specific cues required to reestablish homeostasis - or exhibit their lineage capacity *in vitro*. Our *in vivo* work is an example of this phenomenon in which the transplanted HUCPVCs not only contributed to tissue healing and matrix elaboration themselves, but also recruited resident mouse progenitors to rapidly repair the damaged tissues. Accordingly, rather than displaying unrestricted self-renewal and differentiation, only a few asymmetric cell divisions of the resident MSCs may have been initiated to produce the more restricted tissue-specific progenitors available for regenerating the damaged tissue.

By assaying five mesenchymal lineages, our data demonstrate that the greater the potential a cell has for differentiation, the rarer it is within the mesenchymal compartment. Recent evidence has shown that MSCs arise during an initial transient wave of neuroepithelial differentiation, although these are replaced by MSCs from unknown sources [24]. We have shown here that the muscle lineage is the first to be forfeit, and this reflects embryonic mesenchymal development in which a subsequent wave of paraxial mesoderm development gives rise to initially identical somites that specify into three regions. The dermamyotome and myotome give rise to skeletal muscles of the back, body wall and limbs, while the sclerotome produces the cartilage cells of the vertebrae and ribs that subsequently undergo endochondral ossification. Sequential loss of the chondrogenic and osteogenic lineages inevitably produce the default fibroblastic lineage not only illustrated in the hierarchy of our model, but also observed in adult marrow stroma, the stromal tissue of the other organs of the body and many solid tumors [54], [55].

To date, elucidating the biology of MSCs has been challenging because of the low frequency of MSCs within the heterogeneous bone marrow population. By using a richer, more homogeneous source of primary mesenchymal cells, HUCPVCs, we have dissected the differentiation of MSCs without the confounding effects of the variety of other stem cells, progenitors and terminally differentiated cells present in bone marrow. We have shown that HUCPVCs induce tissue repair in response to injury, and that SCD clonal multipotent parents give rise to a clonal multipotent descendants, satisfying the two properties of stem cells: self-renewal and multilineage differentiation. The ability of these self-renewing progenitors to differentiate along multiple pathways becomes more restricted with the loss of the myogenic, adipogenic, chondrogenic, osteogenic and fibroblastic progenitor lineages respectively, illustrating the hierarchical mechanism of MSC fate decisions. Identifying the molecular cues that arrest or drive this hierarchical cascade will be of considerable importance to our understanding mesenchymal biology.

## Materials and Methods

**Ethical** approval was obtained from both the University of Toronto and Sunnybrook and Women's College Health Sciences Centre, Toronto: written informed consent was obtained and the umbilical cords were donated by consenting full-term caesarian section patients. A total of 57 umbilical cords were used in the experiments described and the number employed for each captioned with the appropriate figure. For additional details see Supplementary Methods S1.

### Colony forming unit (CFU-F) assay

HUCPVCs expanded in culture to 70–90% confluence were harvested with trypsin-EDTA and counted (as described in Supplementary Methods S1). Cells were diluted in supplemented media (SM): 5% fetal bovine serum (FBS) (HyClone, Lot KPF21344), 85% α-MEM, and 10% antibiotics (fungizone, penicillin & streptomycin), and plated at 1, 5, 10, 50, 100, and 500 cells per well of a 6-well culture dish. After incubation for 5–7 days at 37^°^C in 5% humidified CO_2_, the cells were washed with PBS and stained with 0.5% Crystal Violet (Sigma-Aldrich) for 15 min at room temperature. Cells were washed with PBS twice, and visible colonies were counted from 7 different samples. The minimum number of input cells required to produce a single colony determined the CFU-F frequency. This was repeated at every sub-culture until passage 12 (P12).

### *In vivo* analysis of HUCPVC differentiation

P2 HUCPVCs were transduced by lentivirus expressing eGFP driven by the constitutive EF1a promoter (a kind gift from Dr. J. Ellis, MaRS, Toronto, Canada). The eGFP+ cells were expanded in culture to 20×10^6^ cells. 0.5×10^6^ cells were injected in 20 µl of α-MEM into the intrafemoral space of each of 26 NOD-scid mice (Jackson Laboratories), with an additional 10 mice receiving 20 µl of α-MEM (sham). After 2, 4 and 6 weeks, the mice were sacrificed. At each time point, the marrow from 3 mice was flushed and cultured, while the femurs from 4 cell-injected and 3 sham-injected mice were fixed for 24 hours in 10% neutral buffered formalin. The fixed femurs were analyzed by micro computed tomography (micro CT) (General Electric Health Care). A pixel threshold was determined by pixel intensity outside the femur, and software (GE MicroView) was then used to determine all pixels above the threshold as BMD. The fixed femurs were then decalcified in 20% formic acid and embedded in paraffin for sectioning into 4 µm thin sections. Longitudinal sections were obtained through the entire width of the femur. From every 15 sections, 6 serial sections were labeled with Masson's Trichrome, anti-GFP (1∶100, Sigma-Aldrich), biotin-conjugated (Lightning-Link Biotin, Innova Biosciences) mouse-anti-human nuclear-antigen (HuNu) (1∶300, Chemicon) anti-human-osteocalcin (1∶100, Peninsula), anti-human collagen II (1∶100, Chemicon), and anti-human PPARγ (1∶200, Abcam) respectively.

### Clonal isolation of HUCPVCs by sub-single cell seeding

Isolates of male and female P0 HUCPVCs were plated separately for 24 hours, removed from the culture surface by trypsin/EDTA (Supplementary Methods S1), washed and collected as independent suspensions. Each suspension, approximately 5×10^4^ cells (P1), were then passed through a 70 µm cell strainer to ensure single cell suspension, and counted on a ViCell-XR automated cell counter. 5×10^3^ cells from each suspension were mixed together, and an aliquot of the mixed suspension was then diluted in SM to a concentration of 0.5 or 0.2 cells per 50 µl respectively. 50 µl of the mixed suspension was placed into each well of 35×96-well tissue culture plates. After 24 hours, an additional 50 µl of SM was added to each well and the SM was replaced every 5 days. After 10 days in culture, each well of the plates was observed by light microscopy for the presence of cells. Only wells with cells were maintained by replacement of SM every 3 days until they reached 70–90% confluence. The cells were then sub-cultured and seeded (P2) into an individual well of a 6-well culture dish. The media was replaced every 2 days until the cells reached 70–90% confluence, at which point they were sub-cultured and seeded (P3) into individual T-75s, in which the media was replaced every 2 days. Once the cells reached 70–90% confluence, they were removed from the culture surface (P4) by trypsin/EDTA, counted with the Vi-Cell (approximately 1–3× 10^6^ cells) and seeded as required for differentiation assays or stored in liquid N_2_ (Supplementary Methods S1).

### Sub-clonal isolation of HUCPVCs by sub-single cell seeding

P4 clonal HUCPVC populations derived from 0.2 cell/well seeding were obtained as single suspensions by passing them through a 70 µm cell strainer. They were then diluted in SM to 0.2 cells per 50 µl and seeded into 96 well plates (5 plates per clone). The cells were expanded as described above into 6-well plates (P5), and T-75s (P6). Once they reached 70–90% confluence they were removed from the culture surface, and seeded as required for differentiation assays (described below), or stored in liquid N_2_ (described above).

### Double blind analysis of clonal formation by Fluorescent *in-situ* hybridization

As all clones and sub-clones were isolated from equal suspensions of mixed male and female cells, single cell isolation was confirmed by fluorescent *in-situ* hybridization (FISH) analysis for the presence or absence of a Y-chromosome. 5×10^3^ cells from each of the 32 clones and 11 sub-clones, both derived from 0.2 cells/well seeding, were plated into independent wells on 4-well glass chamber slides. One slide was prepared with known controls including, 5×10^3^ mixed male/female, 5×10^3^ male, and 5×10^3^ female (two wells) cells in separate wells. The slides were then provided to an independent FISH analyst who was only given identification of the control wells. Importantly, the analysis was double-blind, as neither the person who plated the cells, nor the independent analyst knew the gender of any of the 43 clones. The CEP Y Sat III-32-112024 DNA FISH probe was obtained from Vysis. Application of the probe was performed following the Vysis protocol, and the cells were counterstained with DAPI. The independent analyst then analyzed the 43 clones for presence/absence of Y-chromosome nuclear localization by fluorescent confocal microscopy. Wells with a Y-chromosome signal equal or less than the negative control (known female cells), or a signal equal or greater than that of the positive control (known male cells) were identified as single-cell derived.

### Differentiation of HUCPVC clones and sub-clones

Each clone was seeded into 12 wells of a 24-well plate. 9 wells were seeded with 10^4^ cells. Of these, 2 of each were induced with medium containing either osteogenic supplemented (OS), adipogenic supplemented (AS) or myogenic supplemented (MS) media (Supplementary Methods S1), and the remaining 3 were treated with SM containing 2% FBS as negative controls. The remaining 3 wells of the plate were seeded with 100, 50 and 10 cells, and treated with SM containing 5% FBS to assess the CFU-F frequency of each clone. Also, 2.5×10^5^ cells from each clone were induced with chondrogenic supplements (CS) in a 15 ml tube (Supplementary Methods S1). RNA from the remaining induced cells was harvested with 0.5 ml Trizol reagent (Invitrogen) for RT-PCR analysis (Supplementary Methods S1 and Table S1).

### Statistical analysis

CFU-F frequencies and proliferation were compared using un-paired 2-tailed t-tests and micro CT data was compared using a paired 2-tailed t-test. T-test values were considered significant with a probability of less than 5% (P<0.05). For FISH analysis, if the admixture of cells of the second donor in each colony is k, then the probability of not finding a mixed colony after identification of 22–54 cells in each colony is (1-k)^22^ to (1-k)^54^. For k = 0.5, the probability of single cell clonal formation was 10^−7^ to 10^−17^.

## Supporting Information

Methods S1 Supplementary Methods
(0.05 MB DOC)

Table S1 Primer sequences used for RT-PCR. - Abbreviations are: lipoprotein lipase (LPL), fast skeletal myosin light chain (FSMLC) and myosin heavy chain (MHC).
(0.05 MB DOC)

Figure S1 (A) indicating their self-renewal capacity, clone ID# and lineage potential. (Clone ID #s correspond to those in Figure 3C). 21 of the 32 parental clones (B) did not produce daughters that survived expansion for lineage analysis. Of these, 12 survived sub-cloning (indicated by √), while 9 did not (indicated by χ).
(0.13 MB DOC)
