# Supplementary material for: Human Mesenchymal Stem Cells Self-Renew and Differentiate According to a Deterministic Hierarchy
Source: PLoS One. 2009 Aug 4;4(8):e6498. doi: 10.1371/journal.pone.0006498 (PMC2714967; doi:10.1371/journal.pone.0006498)
Supplement: Methods S1 — Supplementary Methods (0.05 MB DOC) [file pone.0006498.s001.doc]

**A**

**Supplementary Methods S1**

**Primary culture.** Cells isolated from the perivascular region of the UC were isolated as previously described [1]. Briefly, the UC was cut into 4 cm sections and washed in phosphate buffered saline (PBS -Mg^+^, -Ca^2+^). The epithelium was stripped away with forceps, exposing the underlying matrix. The vessels were then pulled out with their surrounding extracellular matrix intact. The ends of the vessels were tied off with a surgical suture, and placed into a 0.5mg/ml collagenase I (Sigma-Aldrich) digest on a rotisserie in a dry 37^o^C incubator for 18-24 hours. The HUCPVC suspension was then washed with PBS and seeded into a 75cm^2^ tissue culture flask (T-75) with supplemented medium (SM) comprising 5% fetal bovine serum (FBS) (HyClone, Lot KPF21344), 85% α-MEM, and 10% antibiotics (fungizone, penicillin & streptomycin), and incubated at 37^o^C with 5% humidified CO_2_. After 24 h, non-adherent cells were removed. The adherent cells were washed twice with (PBS) (-Mg^+^, Ca^2+^) and fresh SM was added. The medium was replaced every 2 days and the cells were sub-cultured at 70-90% confluence.

**Sub-culture and cell proliferation.** Upon reaching 70-90% confluence as estimated by phase microscopy, the cells were washed with PBS and removed from the culture dish surface by 5 ml 0.02% trypsin and 200nM EDTA (Gibco) for 5 min at 37^o^C. The total number of live cells was ascertained with the use of a ViCell-XR automated cell counter (Beckman Coulter). To expand the cells through successive passages, they were plated at 4,000 cells/cm^2^, allowed to reach 70-90% confluence and sub-cultured as described. Proliferation rate of the cells was determined using the formula N=N_0_e^kt^, from 9 different samples.

**Cryopreservation of cells.** Residual cells from sub-culture (0.5 to 5x10^6^) were cryopreserved using 80% FBS and 20% dimethyl sulphoxide (DMSO) (Sigma-Aldrich), and transferred into 1.5 ml polypropylene cryo-vials. The vials were placed into a -70^o^C freezer overnight, and transferred the following day to a liquid N_2_ (-196^o^C) freezer for long-term storage.

**Clonal isolation of HUCPVCs by limiting dilution.** We previously determined the CFU-F frequency of primary HUCPVCs to be 1:300^1^. Then, 300 passage 0 (P0) HUCPVCs were seeded into individual wells of 6-well tissue culture plates by limiting dilution. After 5-7 days, wells with single distinct colonies as determined by light microscopy were marked for isolation. The colonies were isolated by one of two methods. The first method was use of a ‘cloning cylinder.’ A sterile 8 mm diameter polystyrene cylinder was dipped in sterile vacuum grease, and placed around the pre-marked colony. The colony was then washed and removed from the culture dish with the use of 100μl of trypsin/EDTA (as described above), transferred to an individual well of a new 6-well plate with fresh SM. Alternatively, the ‘mechanical removal’ method was used. Distinct colonies were marked on the underside of the well with a permanent marker. A cell-scraper was then used to wipe the surface of the entire well with the exception of the marked colony. The well was then washed twice with PBS and replaced with fresh SM. Light microscopy was used to determine that no other adherent cells outside of the colony remained in the well. In both methods, the colony was then allowed to proliferate until 70-90% confluence before sub-culture and seeding into a T-75.

**Clonal isolation of HUCPVCs in agarose.** NuSieve GTG low-melting temperature agarose (BioWhittaker) was made up at the following concentrations: 2.5%, 2.0%, 1.5%, 1.0% and 0.5%, in 85% α-MEM, 5% FBS and 10% antibiotics. 300 HUCPVCs were then cultured either on the surface of the agarose, or in suspension. Any growing colonies were observed after 10 days of culture.

**Clonal isolation of HUCPVCs by MoFlo®.** P0 HUCPVCs were plated for 24 hours, and removed from the culture surface by trypsin/EDTA (described above). The cells (approx. 5x10^4^) were labeled with propidium iodide (Invitrogen). A MoFlo® fluorescent activated cell sorter (Dako) was calibrated to deposit 1 live cell per well in 96-well tissue culture plates with 100μl SM in each well. To determine rigorous single-cell seeding, cells were only seeded when the drops immediately preceding and succeeding it were vacant.

**Clonal isolation of HUCPVCs by single cell seeding.** Isolates of male and female P0 HUCPVCs were plated separately for 24 hours, removed from the culture surface by trypsin/EDTA (described above), washed and collected as independent suspensions. Each suspension, approximately 5x10^4^ cells (P1), were then passed through a 70μm cell strainer to ensure single cell suspension, and counted on the ViCell-XR automated cell counter. 5x10^3^ cells from each suspension were mixed together, and an aliquot of the mixed suspension was then diluted in SM to a concentration of 1 cell per 50μl. 50μl of the mixed suspension was placed into each well of 35 x 96-well tissue culture plates. After 24 hours, an additional 50μl of SM was added to each well and the SM was replaced every 5 days. After 10 days in culture, each well of the plates was observed by light microscopy for the presence of cells. Only wells with cells were maintained by replacement of SM every 3 days until they reached 70-90% confluence. The cells were then sub-cultured and seeded (P2) into an individual well of a 6-well culture dish. The media was replaced every 2 days until the cells reached 70-90% confluence, at which point they were sub-cultured and seeded (P3) into individual T-75s, in which the media was replaced every 2 days. Once the cells reached 70-90% confluence, they were removed from the culture surface (P4) by trypsin/EDTA, counted with the Vi-Cell (approximately 1-3x10^6^ cells) and seeded as required for differentiation assays (described below), or stored in liquid N_2_ (described above).

**Lineage induction and analysis: Osteogenic:** Induced cells were placed in osteogenic supplemented (OS) media comprising 2% FBS, 88% α-MEM, and 10% antibiotics, supplemented with 10nM dexamethasone (Sigma-Aldrich), 5mM β-glycerophosphate (Sigma-Aldrich), and 50μg/ml L-ascorbic acid (Sigma-Aldrich). The OS medium was replaced every 2 days until bone nodules were observed (usually after 7-14 days), at which point the cultures were treated with SM containing 9μg/ml tetracycline (Sigma-Aldrich), and visualized 24 hours later for fluorescent foci of mineralization. One well was then fixed in 3.7% formalin for alkaline phosphatase and Von Kossa staining (see below) along with its uninduced negative control. **Chondrogenic:** 2.5x10^5^ cells were placed into a 15ml tube and centrifuged at 1150rpm for 5 minutes to form a cell pellet. The media was removed and replaced with 0.5ml chondrogenic supplemented (CS) media comprising 90% α-MEM, 10% antibiotics, and 10ng/ml transforming growth factor- β1 (TGF-β1) (Chemicon). The CS medium was replaced every 4 days for 21 days. The chondrogenic pellets were then removed from the CS medium and cut in half with a scalpel blade. One half was fixed in 3.7% formalin for Alcian blue staining and collagen II immunostaining with mouse-anti-human collagen II (Chemicon), while RNA from the remaining half was harvested with 0.5ml Trizol reagent for rtPCR analysis (described below). **Adipogenic:** Induced cells were placed in adipogenic supplemented (AS) media comprising 87% α-MEM, 10% antibiotics, 3% FBS, 33μM biotin (Sigma-Aldrich), 17μM Pantothenate (Sigma-Aldrich), 5μM Rosiglitazone (Cayman Chemical), 100nM bovine insulin (Sigma-Aldrich), 1μM dexamethazone (Sigma-Aldrich), and 200μM isobutyl methylxanthine (Sigma-Aldrich). The AS medium was replaced every 2 days until colonies of adipocytes could be observed. One well was stained with Oil Red O (Sigma-Aldrich) along with its uninduced negative control. **Myogenic:** Induced cells were placed in myogenic supplemented (MS) media comprising 88% α-MEM, 10% antibiotics, 2% horse serum (Gibco, Lot 480116), 1 nM dexamethasone (Sigma-Aldrich), and 2μM hydrocortisone (Sigma-Aldrich). The culture medium was replaced every 2 days until multinucleated myotubes could be observed (28 days). One well was then fixed in 3.7% formalin for immunostaining staining along with its uninduced negative control. For immuno-histochemistry, fixed cells were first permeabilized with 100% methanol for 2 minutes, and blocked with 10% FBS/PBS for 1 hour. The cells were then incubated with 1μl each of rabbit-anti-human MyoD primary antibody (Santa Cruz biotech) and mouse-anti-human fast skeletal myosin light chain primary antibody (Sigma-Aldrich) overnight, then washed thoroughly. Goat-anti-rabbit Alexa Fluor 488 secondary antibody (Molecular Probes) and goat-anti-mouse Alexa Fluor 555 secondary antibody (Molecular Probes) were then incubated with the cells for 1 hour and washed thoroughly. As a negative control, the primary antibody was excluded. A Leica DMIRE2 inverted fluorescent microscope (Wetzlar, Germany) was used to visualize fluorescence at 519nm (green) and 565nm (red) wavelengths.

**Alkaline phosphatase staining**

Cells were fixed in 10% cold Neutral Formalin Buffer (NFB) for 15 minutes, after which the buffer was removed, the dish rinsed once with distilled water, and left in water for 15 minutes. While waiting, fresh substrate was prepared by combining 0.005g Naphthol AS MX-PO_4_ (Sigma #N-2125, Lot 38H5232), 200 μl DMF (N,N-Dimethylformamide), 25 ml Tris-HCl (MW = 157.6, 0.2 M, pH 8), 25 ml distilled water, and 0.03g Red Violet LB salt (Sigma #F-3381, Lot 106H3685). The substrate was filtered with Whatman’s No.1 filter paper immediately prior to addition to the dishes to be stained. The dishes were incubated for 45 minutes at room temperature, then rinsed in distilled water 3-4 times and observed for ALP expression by the presence of a red-orange precipitate.

**Von Kossa staining**

Dishes were then stained with 2.5% silver nitrate for 30 min, after which the silver nitrate was removed and rinsed with distilled water 3 times. Prior to examination or drying the dish, the colour of the mineralized nodules was deepened by adding sodium carbonate formaldehyde to the dish for 30s – 2min. The sodium carbonate formaldehyde was removed and the dish was rinsed with slowly running tap water. The stained cells were rinsed in tap water for 1 hour and then rinsed again and covered in tap water for counting or set to dry.

**RNA Isolation and Reverse Transcription (RT).** Total RNA was isolated from cell cultures by treatment with 0.5ml Trizol reagent (Invitrogen) Four micrograms of total RNA was used in each RT reaction with specific primer pairs (Supplementary Table S1) used to amplify first strand cDNA. The amplified PCR product was fractionated on a 1.2% agarose gel and visualized by ethidium bromide staining.
