# Supplementary material for: Human Mesenchymal Stem Cells Self-Renew and Differentiate According to a Deterministic Hierarchy
Source: PLoS One. 2009 Aug 4;4(8):e6498. doi: 10.1371/journal.pone.0006498 (PMC2714967; doi:10.1371/journal.pone.0006498)
Supplement: Table S1 — Primer sequences used for RT-PCR. - Abbreviations are: lipoprotein lipase (LPL), fast skeletal myosin light chain (FSMLC) and myosin heavy chain (MHC). (0.05 MB DOC) [file pone.0006498.s002.doc]

| Gene | F/R | Sequence (5’-3’) | Anneal (°C) |
| --- | --- | --- | --- |
| Runx2 | F | AAAGTTACAGTAGATGGACCTCGGG | 58.8 |
|  | R | ATTCGTGGGTTGGAGAAGCG |  |
| Collagen IA1 | F | ATGCCTGGTGAACGTGGT | 57.8 |
|  | R | AGGAGAGCCATCAGCACCT |  |
| Osteocalcin | F | TGACGAGTTGGCTGACCACATC | 57.2 |
|  | R | GCAAGGGGAAGAGGAAAGAAGG |  |
| Osteopontin | F | ACGCCGACCAAGGAAAACTC | 56.2 |
|  | R | GTCCATAAACCACACTATCACCTCG |  |
| Sox9 | F | TTCAGAGCAAGCGTGGAGGATG | 52.6 |
|  | R | AATGTTTCCCAGCAGCACCG |  |
| Collagen II | F | AACCAGATTGAGAGCATCCGC | 58.6 |
|  | R | CGATAACAGTCTTGCCCCACTTAC |  |
| Aggrecan | F | GGTGAAGACTTTGTGGACATCCC | 58.6 |
|  | R | CATTCTCAACCTCAGCGAAGGC |  |
| LPL | F | TGGCTGGACGGTAACAGGAATG | 56.8 |
|  | R | CATCATCAGGAGAAAGACGACTCG |  |
| MyoD | F | TTGCCACAACGGACGACTTC | 57.5 |
|  | R | AGTGCTCTTCGGGTTTCAGGAG |  |
| Myf5 | F | TTTGGGGACGAGTTTGTGCC | 59.9 |
|  | R | TAGTTCTCCACCTGCTCTCTCAGC |  |
| Desmin | F | CAGCCAACAAGAACAACGACG | 59.2 |
|  | R | GTATGGACCTCAGAACCCCTTTG |  |
| FSMLC | F | TGTGAAGAATGAGGAGTTGGATGC | 52.2 |
|  | R | CAGGAAGACGGTGAAGTTGATGG |  |
| MHC | F | CAAATCATCAGTGCCAACCCC | 56.4 |
|  | R | TACTCATTGCCGACCTTGACCC |  |
