# Supplementary figures and images for: Human Mesenchymal Stem Cells Self-Renew and Differentiate According to a Deterministic Hierarchy
Source: PLoS One. 2009 Aug 4;4(8):e6498. doi: 10.1371/journal.pone.0006498 (PMC2714967; doi:10.1371/journal.pone.0006498)

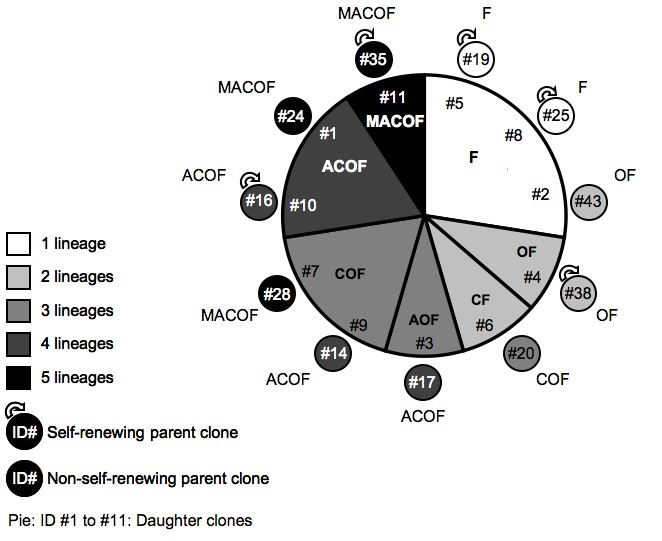


**A**

**B**

Supplement: Figure S1 — (A) indicating their self-renewal capacity, clone ID# and lineage potential. (Clone ID #s correspond to those in Figure 3C). 21 of the 32 parental clones (B) did not produce daughters that survived expansion for lineage analysis. Of these, 12 survived sub-cloning (indicated by √), while 9 did not (indicated by χ). (0.13 MB DOC) [file pone.0006498.s003.doc]
